# Impacts of Watershed Characteristics and Crop Rotations on Winter Cover Crop Nitrate-Nitrogen Uptake Capacity within Agricultural Watersheds in the Chesapeake Bay Region

**DOI:** 10.1371/journal.pone.0157637

**Published:** 2016-06-28

**Authors:** Sangchul Lee, In-Young Yeo, Ali M. Sadeghi, Gregory W. McCarty, W. Dean Hively, Megan W. Lang

**Affiliations:** 1 Department of Geographical Sciences, University of Maryland, College Park, Maryland, United States of America; 2 School of Engineering, The University of Newcastle, Callaghan NSW, Australia; 3 USDA-ARS, Hydrology and Remote Sensing Laboratory, Beltsville, Maryland, United States of America; 4 USGS, Eastern Geographic Science Center, Reston, Virginia, United States of America; Agricultural Research Service, UNITED STATES

## Abstract

The adoption rate of winter cover crops (WCCs) as an effective conservation management practice to help reduce agricultural nutrient loads in the Chesapeake Bay (CB) is increasing. However, the WCC potential for water quality improvement has not been fully realized at the watershed scale. This study was conducted to evaluate the long-term impact of WCCs on hydrology and NO_3_-N loads in two adjacent watersheds and to identify key management factors that affect the effectiveness of WCCs using the Soil and Water Assessment Tool (SWAT) and statistical methods. Simulation results indicated that WCCs are effective for reducing NO_3_-N loads and their performance varied based on planting date, species, soil characteristics, and crop rotations. Early-planted WCCs outperformed late-planted WCCs on the reduction of NO_3_-N loads and early-planted rye (RE) reduced NO_3_-N loads by ~49.3% compared to the baseline (no WCC). The WCCs were more effective in a watershed dominated by well-drained soils with increased reductions in NO_3_-N fluxes of ~2.5 kg N·ha^-1^ delivered to streams and ~10.1 kg N·ha^-1^ leached into groundwater compared to poorly-drained soils. Well-drained agricultural lands had higher transport of NO_3_-N in the soil profile and groundwater due to increased N leaching. Poorly-drained agricultural lands had lower NO_3_-N due to extensive drainage ditches and anaerobic soil conditions promoting denitrification. The performance of WCCs varied by crop rotations (i.e., continuous corn and corn-soybean), with increased N uptake following soybean crops due to the increased soil mineral N availability by mineralization of soybean residue compared to corn residue. The WCCs can reduce N leaching where baseline NO_3_-N loads are high in well-drained soils and/or when residual and mineralized N availability is high due to the cropping practices. The findings suggested that WCC implementation plans should be established in watersheds according to local edaphic and agronomic characteristics for reducing N leaching.

## Introduction

The Chesapeake Bay (CB) is the largest and most productive estuary in the United States (US). Eleven major rivers flow into the bay's 166,000 km^2^ drainage basin. Despite significant restoration efforts, the health of the bay has continued to deteriorate primarily as a result of loading of nutrients and sediments from agricultural land [1]. Nitrate-N in soil and groundwater can be stored for a relatively long time before discharging to streams. The lag time between the implementation of land-based Best Management Practice (BMP) and the realization of nutrient reductions can cause uncertainty regarding the effect of BMPs [1]. Nitrogen-riched groundwater on the eastern shore of the Chesapeake Bay Watershed (CBW) has significant implication on the bay ecosystem, as groundwater in the Mid-Atlantic Coastal Plain contributes a large portion of stream flow (~70%) [1]. Previous studies have demonstrated that NO_3_-N flux in groundwater is spatially related to the proportion of agricultural land in coastal watersheds [2]. For example, Ator and Denver (2012) [3] reported that a watershed dominated by agriculture exported 9-fold more NO_3_-N flux from groundwater to streams than other watersheds dominated by non-agricultural lands in coastal regions.

Winter cover crops (WCCs) have been identified as a potentially important BMP for reduction of NO_3_-N loads in the CBW [4]. Specifically, in the coastal plain of the CBW, in-stream NO_3_-N concentration during the winter season from October (after harvest of summer crops) to the following March (before planting of summer crops) can be very high [5]. An earlier study by Fisher et al. (2010) [2] showed that winter NO_3_-N concentration can be nearly five times greater than that during the late summer. During the winter season, rising groundwater level can also increase NO_3_-N concentration. This collectively leads to higher in-stream NO_3_-N loadings to the bay [2, 6]. The WCCs can reduce residual soil N after harvest of summer crops and therefore N leaching by converting it to crop biomass N [7, 8]. Therefore, the WCCs have become a promising BMP for improving water quality in this region. Because of their potential to improve water quality, federal and state government agencies are providing technical assistance and financial incentives to local farmers to encourage planting WCCs in the agricultural lands within the watershed.

The potential of WCCs to reduce NO_3_-N loads to the bay, however, has not been fully assessed at the watershed scale considering different land characteristics and agricultural practices. Field studies have demonstrated reduction in soil NO_3_-N concentration after planting WCCs, but they were limited to plot-scale studies [6, 8]. Findings from these field studies do not necessarily reflect the long-term impacts of WCCs at the watershed scale and have limited ability to evaluate the performance of WCCs under various soil and weather conditions. Recently, Yeo et al. (2014) [9] conducted a watershed-scale assessment of WCC efficiency in a small agricultural watershed. They, however, did not demonstrate the effects of drainage condition of soils and agricultural practices on WCC performance. Hydrogeological conditions and agricultural practices including crop rotations can affect NO_3_-N loads and the performance of WCC. For instance, well-drained soils characterized by greater infiltration rates promote the downward movement of water and N leaching. With aerobic soils and aquifer conditions, NO_3_-N tends to remain stable in groundwater and can later be transported to streams [3]. Meanwhile, poorly-drained soils characterized by lower infiltration rates have water-saturated (i.e., anaerobic) conditions, favorable to denitrification [10]. Agricultural production on poorly-drained soils often requires extensive artificial drainage system, which can shorten flow pathways and reduce the amount of time it takes for NO_3_-N to reach nearby streams [2, 4]. In addition, soil residual N after harvest of previous crops can vary by crop species [11]. Mineralization of crop residue and soil organic matter can also affect NO_3_-N concentration in the soil [12]. The chemical composition (i.e., C/N ratio) and the amount of crop residue returned to the soil can also affect NO_3_-N [12]. For example, Kaboneka et al. (1997) [13] reported that soybean residue released greater N from mineralization than corn and wheat due to higher N concentration.

Despite similar climatic conditions, agronomic practices, and watershed size, in-stream NO_3_-N concentrations (and therefore NO_3_-N loads) are quite different in the Tuckahoe Creek and Greensboro watersheds, two adjacent agricultural watersheds located in the coastal plain of the CBW. The goal of this study was to assess the long-term (2001–2008) impact of WCC practices, watershed characteristics, and crop rotations on catchment hydrology and NO_3_-N loads in the Tuckahoe Creek and Greensboro watersheds using the Soil and Water Assessment Tool (SWAT) model and statistical methods. Process-based water quality models, such as the SWAT, have been shown to be promising tools for the evaluation of long-term BMP effectiveness on water quality improvement at the watershed scale [14] and development of site-specific management plans. The SWAT model was applied to both watersheds under multiple WCC implementation scenarios (e.g., crop species and timing). Using this model, we 1) investigated how different soil and land use characteristics affect the generation and transport of NO_3_-N fluxes, and the NO_3_-N removal efficiency of WCC at the watershed and cropland scale, and 2) evaluated the effects of crop rotations on soil N and NO_3_-N concentration. Following the SWAT modeling, multiple statistical analyses were performed to assess if the simulation outputs under the WCC scenarios were statistically different from those under the baseline scenario (no WCC). We integrated various time series geospatial data layers and county statistics to develop more realistic scheduling and placement of crop rotations, and used WCC reports to develop different WCC management scenarios. Extending the methodology used by Yeo et al. (2014) [9], the plant growth model embedded in the SWAT was further calibrated to accurately simulate WCC biomass and nutrient uptake.

## Materials and Methods

### Study area

**Fig 1:**
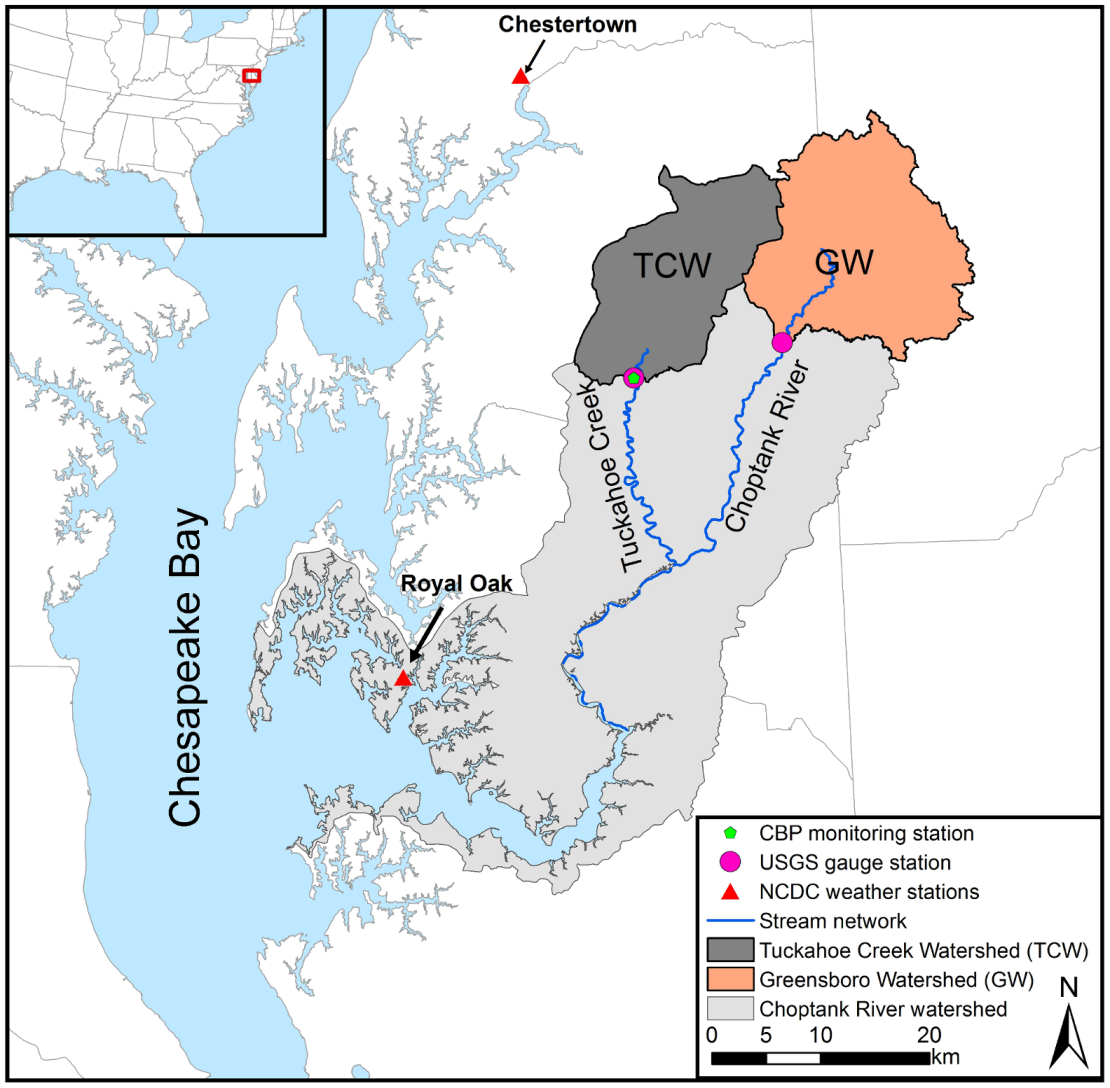
The location of the Tuckahoe Creek Watershed and Greensboro Watershed near Chesapeake Bay. Reprinted from Yeo et al. (2014) [9] under a CC BY license, with permission from the authors of the article, original copyright 2014.

This study was undertaken in two adjacent watersheds defined by U.S. Geological Survey (USGS) gauge stations at Tuckahoe Creek near Ruthsburg (USGS#01491500) and the Choptank River near Greensboro (USGS#01491000) which are referred to as the Tuckahoe Creek Watershed (TCW, ~220.7 km^2^) and Greensboro Watershed (GW, ~290.1 km^2^), respectively (Fig 1). They are located on the headwaters of the Choptank River watershed in the coastal plain of the CBW (Fig 1). The Choptank River watershed is a U.S. Department of Agriculture (USDA) Conservation Effects Assessment Project (CEAP) Benchmark Watershed [4]. Due to high nutrient levels (in particular NO_3_-N) in addition to sediments and bacteria, the Choptank River is listed as “impaired” by the U.S. Environmental Protection Agency (EPA) under Section 303(d) of the 1972 Clean Water Act and is subject to extensive monitoring [4].

**Fig 2:**
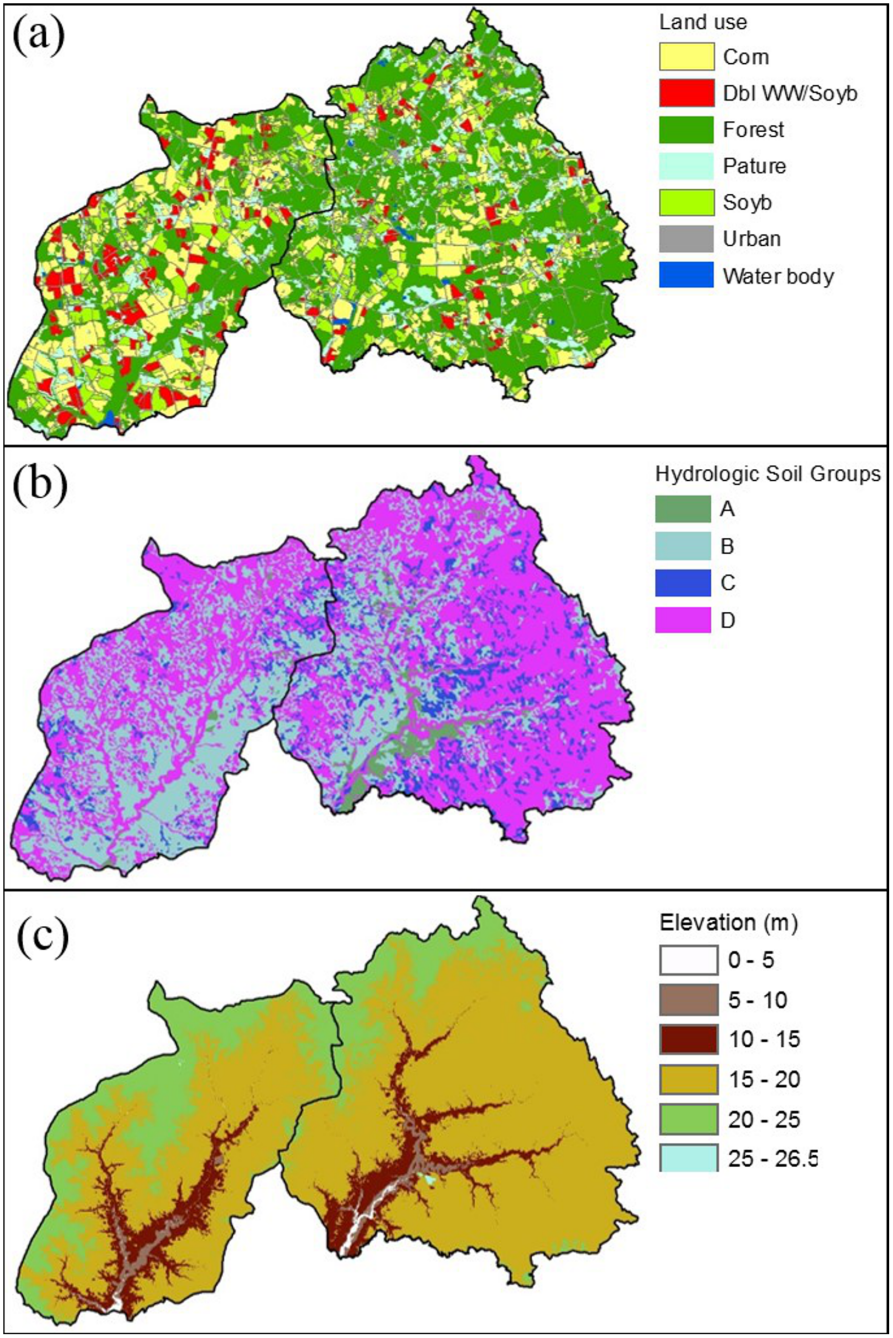
The physical characteristics of the Tuckahoe Creek Watershed (left) and Greensboro Watershed (right); (a) land use, (b) hydrologic soil groups, and (c) elevation. Note: Dbl WW/Soyb stands for double crops of winter wheat and soybean in a year. Hydrologic soil groups (HSGs) are characterized as follows: Type A- well-drained soils with 7.6–11.4 mm/hr (0.3–0.45 inch/hr) water infiltration rate; Type B—moderately well-drained soils with 3.8–7.6 mm/hr (0.15–0.30 inch/hr) water infiltration rate; Type C—moderately poorly-drained soils with 1.3–3.8 mm/hr (0.05–0.15 in/hr) water infiltration rate; Type D—poorly-drained soils with 0–1.3 mm/hr (0–0.05 inch/hr) water infiltration rate [17].

The two adjacent watersheds have very different characteristics in terms of soil properties and land use (Fig 2 and S1 Table). In TCW, major land uses are agriculture (54.0%) and forestry (32.8%) dominated (56.1%) by well-drained soils (Hydrologic Soil Group, HSG-A&B) where 69.5% of the area are under croplands. In comparison, GW has a higher percentage of forest (48.3%) and a lower percentage of agricultural area (36.1%). A large portion of soils in GW (74.5%) is poorly-drained (HSG-C&D) where 67.2% of croplands are located [15]. In this region, drainage ditches have been established mostly on poorly-drained croplands for crop production. As a result, drainage ditches are widespread in GW compared to TCW [4]. Artificial subsurface drainage (e.g., tile drains) is less common in both watersheds. In general, well-drained soils have higher water infiltration capacity, produce little surface runoff, and facilitate the downward movement of water [16]. In comparison, poorly-drained soils have lower water infiltration capacity, substantial surface runoff, and limited water percolation [16]. Given the contrasting soil properties and land use in two watersheds, NO_3_-N is expected to be lost mainly through leaching in TCW and near-surface runoff in GW [4]. Nitrate-N concentration in stream flow has been shown to vary with cropland soil properties according to regional water quality studies [5]. Water quality records showed that the in-stream NO_3_-N concentration in TCW is nearly two times higher than GW [4]. It is expected that NO_3_-N removal by WCC would be greater in TCW due to greater area under crop production than GW.

### SWAT watershed process model

The SWAT model has been widely used to evaluate water quality and assess effectiveness of BMPs [17]. Major SWAT components include weather, hydrology, soil temperature, sedimentation, nutrients, pesticides, pathogens, plant growth, and land management [17]. The model operates by partitioning a watershed into sub-watersheds, and then into hydrologic response units (HRUs) based on unique combinations of soil, land-use, and slope characteristics. Fluxes of water, sediment, nutrient, and other constituents of interest are simulated and computed at the HRU level and then aggregated to the sub-watershed and ultimately to the larger watershed through routing processes. Water balance within an HRU is determined based on precipitation, surface runoff, evapotranspiration (ET), percolation, and groundwater recharge. Surface runoff is calculated using the Soil Conservation Service (SCS) Curve Number (CN) method [17]. A daily CN is calculated based on soil permeability, land use, and antecedent soil water conditions. Once water enters the soil layers, it can evaporate, be taken up and transpired by plants, and flow into a surface water body through subsurface lateral flow, or percolate into groundwater through the vadose zone (i.e., unsaturated zone) between the bottom of soil layers (i.e., root zone) and the top of groundwater. Percolation, or downward flow, occurs when a soil layer exceeds its field capacity and the layer below is not saturated. The water entering the vadose zone flows into groundwater and its travel time in the vadose zone varies by the depth to the water table and hydraulic properties of the vadose and groundwater zones. The water in groundwater is partitioned into shallow groundwater, groundwater contribution to stream flow (i.e., groundwater flow), water discharge to the overlaying unsaturated zone, and deep groundwater.

The N cycle is fully simulated in the SWAT model. Nitrogen is normally added by fertilizer, crop residue, N fixation, and wet and dry deposition, and removed by plant uptake, leaching, volatilization, denitrification, and surface runoff. Nitrate-N in the soil results from five processes: (1) nitrification (conversion of NH_4_-N to NO_3_-N), (2) addition of manure and N fertilizer, (3) mineralization of soil organic N, (4) biological N fixation, and (5) mineralization of crop residue N.

### Input data

The SWAT model requires detailed information on the climate, soils, and land use for the study site (Table 1). Daily precipitation and temperature were downloaded from the National Oceanic Atmospheric Administration (NOAA) National Climate Data Center (NCDC) at Chestertown and Royal Oak (USC00181750 and USC00187806, respectively) (Fig 1). Other climatic variables, such as daily solar radiation, relative humidity, and wind speed, were generated by the weather generator (WXGEN) embedded in the SWAT [17] due to data unavailability. Since the two watersheds are located side-by-side, we assumed that similar climate conditions prevail for both watersheds. By using the same climate inputs to both watersheds, we evaluated the impacts of soil properties and crop rotations on WCC performance. Monthly stream flow data for both watersheds were downloaded from USGS gauge stations on the Tuckahoe Creek near Ruthsburg (USGS#01491500) and the Upper Choptank River near Greensboro (USGS#01491000) (Fig 1). Nitrate-N concentration grab sample data were provided by the Chesapeake Bay Program (CBP, TUK#0181) for TCW and by USGS (USGS#01491000) for GW. These data were extrapolated to monthly NO_3_-N loads using the USGS LOAD ESTimator (LOADEST) program [18] which has been widely used to estimate continuous water quality information from grab samples [19].

**Table 1:** The list of input data.

Data	Source	Description	Year
DEM	MD-DNR	LiDAR-based 2 meter resolution	2006
Land use	USDA-NASS	Cropland Data Layer (CDL)	2008 ~ 2012
	MRLC	National Land Cover Database (NLCD)	2006
	USDA-FSA-APFO	National Agricultural Imagery Program digital Orthophoto quad imagery	1998
	US Census Bureau	TIGER road map	2010
Soils	USDA-NRCS	Soil Survey Geographical Database (SSURGO)	2012
Climate	NCDC	Daily precipitation and temperature	1999 ~ 2008
Stream flow	USGS	Monthly stream flow	2001 ~ 2008
Water quality	USGS and CBP	Daily grab NO_3_-N samples	2001 ~ 2008

Note: MD-DNR stands for Maryland Department of Natural Resources. USDA-FSA-APFO stands for USDA-Farm Service Agency-Aerial Photography Field Office.

A soil map was prepared based on the USDA Natural Resources Conservation Service (NRCS) Soil Survey Geographic Database (SSURGO). Topography was delineated by resampling a 1 m Light Detection and Ranging (LiDAR)-based Digital Elevation Model (DEM, processed by the USDA-Agricultural Research Service (ARS) at Beltsville, Maryland) to 10 m using nearest-neighbor interpolation, since finer-scale DEMs have been found to overestimate slope parameter values in the SWAT [20]. The land use map and the scheduling of crop rotations were generated using 2008–2012 data from the USDA-National Agriculture Statistics Service (NASS) Cropland Data Layer (CDL), the Multi-Resolution Land Characteristics (MRLC) Consortium-National Land Cover Database (NLCD), digitized boundaries of agricultural fields, and the U.S. Census Bureau Topologically Integrated Geographic Encoding and Referencing (TIGER) road map. We assumed that there was no significant change in crop rotations (see S1 Fig and S2 Table) between the period of our SWAT simulation (1999–2008) and the period of USDA-NASS CDLs coverage (2008–2012). The boundaries of agricultural fields were digitized based on National Agricultural Imagery Program Digital Orthophoto Quad Imagery (1:12,000) and other land use types were delineated using the NLCD. The TIGER road map buffered out by 40 m was intersected with land use maps to better represent urban (i.e., impervious land cover) areas. For each agricultural boundary, the major crop types and their rotations were identified. From the resulting sequence of observed crop rotations, we applied five most frequent crop rotations to the SWAT simulation years used in this study (S2 Table). The placement and sequence of crop rotations for the two watersheds are provided in S1 Fig and S2 Table. Corn, soybean, and double crop winter wheat/soybean were the most frequently grown crops in this region (S1 Fig).

Detailed agronomic management information for field crops was collected through literature reviews and extension agents familiar with the watersheds. Based on collected data, we established the most representative management practices for these regions. We reduced N fertilization rate by 45 kg N·ha^-1^ for corn after soybean compared with corn after corn, due to N credit from soybean residue, based on local expert knowledge (Table 2). The specific agronomic practices, including the timing of planting and harvest of summers crops and the amount and type of fertilizer applications (Table 2 and S2 Fig), were provided by personal communication with R. J. Kratochivil (Assoc. Prof., Dept. of Plant Science & Landscape Architecture, University of Maryland, MD) in May 2014.

**Table 2:** The management schedules for baseline and winter cover crop scenarios.

Baseline scenario (no winter cover crop)			
Crop	Planting	Fertilizer	Harvest
Corn (after corn)	Apr. 30 (no-till)	157 kg N·ha^-1^ (140 lb N·acre^-1^) of poultry manure on Apr. 20 45 kg N·ha^-1^ (40 lb N·ha^-1^) of sidedress 30% UAN on Jun. 7	Oct. 3
Corn (after Soybean and Double crop soybean)	Apr. 30 (no-till)	124 kg N·ha^-1^ (110 lb N·acre^-1^) of poultry manure on Apr. 20 34 kg N·ha^-1^ (30 lb N·ha^-1^) of sidedress 30% UAN on Jun. 7	Oct. 3
Soybean	May 20 (no-till)		Oct. 15
Double crop winter wheat (Dbl WW)	Oct. 10	34 kg N·ha^-1^ (30 lb N·acre^-1^) of sidedress 30% UAN on Oct. 8 45 kg N·ha^-1^ (40 lb N·acre^-1^) of sidedress 30% UAN on Mar. 1 67 kg N·ha^-1^ (60 lb N·acre^-1^) of sidedress 30% UAN on Apr. 5	Jun. 27
Double crop soybean (Dbl Soyb)	Jun. 29		Nov. 1
Winter cover crop scenario			
Crop	Planting	Fertilizer	Harvest
Corn (after corn)	Apr. 30 (no-till)	157 kg N·ha^-1^ (140 lb N·acre^-1^) of poultry manure on Apr. 20 45 kg N·ha^-1^ (40 lb N·acre^-1^) of sidedress 30% UAN on Jun. 7	Oct. 1 or 30
Corn (after Soybean and Double crop soybean)	Apr. 30 (no-till)	124 kg N·ha^-1^ (110 lb N·acre^-1^) of poultry manure on Apr. 20 34 kg N·ha^-1^ (30 lb N·ha^-1^) of sidedress 30% UAN on Jun. 7	Oct. 1 or 30
Soybean	May 20 (no-till)		Oct. 1 or 30
Double crop winter wheat (Dbl WW)	Oct. 10	34 kg N·ha^-1^ (30 lb N·acre^-1^) of sidedress 30% UAN on Oct. 8 45 kg N·ha^-1^ (40 lb N·acre^-1^) of sidedress 30% UAN on Mar. 1 67 kg N·ha^-1^ (60 lb N·acre^-1^) of sidedress 30% UAN on Apr. 5	Jun. 27
Double crop soybean (Dbl Soyb)	Jun. 29		Nov. 1
Winter cover crop	Oct. 3 & Nov. 2		Mar. 31 (Killing)

Note: The typical nitrogen content for poultry manure is 2.8% [9].

Guidelines for winter cover crop implementation practices including recommended planting dates and species were developed by the Maryland Agricultural Cost Share (MACS) cover crop program [21]. The MACS offers varying incentives for planting winter cover crops, primarily depending on species and planting date [21]. Farmers can gain more incentives when they plant WCCs earlier because early-planted WCCs can reduce residual soil N content more effectively than late-planted WCCs. Based on the guideline of the MACS, WCC expert knowledge, and country-level statistics, we established typical early (Oct. 1) and late (Oct. 30) planting dates for WCCs, two harvest dates of summer crops corresponding to the two WCC planting dates (Table 2), and three most commonly used planting species (i.e., wheat (*Triticum aestivum* L.), winter barley (*Hordeum vulgare* L.), and rye (*Secale cereale* L.) in our WCC scenarios (Table 3). In our SWAT model, summertime agronomic practices were kept the same for both baseline (no WCC) and WCC scenarios, but management differed during winter seasons when WCCs were planted on fallow croplands. WCCs were planted after harvesting summer crops, either in the beginning of October (early planting) or November (late planting), and were killed at the beginning of the subsequent growing season (Mar. 31) (Table 2). It was assumed that WCCs could not be placed concurrently with double crop winter wheat, but they could be planted late after harvest of double crop soybean. We did not predict differences in N uptake by summer crops due to shorter versus longer season varieties caused by the two WCC planting dates. The SWAT modeled the maturity for summer crops occurring by the end of September and demonstrated that both early- and late-harvested summer crops (due to two WCC planting dates) have similar biomass and N uptake by the end of September.

**Table 3:** Winter cover crop scenarios.

Scenario	Cover crop species	Planting timing	Abbreviations
**1**	None	N/A	Baseline
**2**	Winter wheat	Early planting (Oct 3)	WE
**3**	Winter Barley	Early planting (Oct 3)	BE
**4**	Rye	Early planting (Oct 3)	RE
**5**	Winter wheat	Late planting (Nov 1)	WL
**6**	Winter Barley	Late planting (Nov 1)	BL
**7**	Rye	Late planting (Nov 1)	RL

Note that the evaluation of WCC effects on water and NO_3_-N budgets was carried out at two spatial scales. First the overall impacts of different WCC species and planting dates were assessed at the watershed scale. Then the effects of WCC placement on different soils and interaction with N availability based on modeled crop rotations were analyzed at the cropland scale, using the SWAT model results for only cropland areas.

### Calibration and validation of the SWAT model

The SWAT simulations were conducted at a monthly time step over the period of 1999–2008. Cumulative daily water and NO_3_-N fluxes delivered to streams by surface runoff, lateral flow, groundwater flow, and percolation/leaching over a month were represented as monthly outputs. The simulations included a 2-year warm-up (1999–2000), 5-year calibration (2001–2005), and 3-year validation (2006–2008) time periods. The model was carefully calibrated based on information from previous SWAT modeling studies in the region [9] and literature values (Table 4). We first calibrated the parameters pertaining to stream flow and then NO_3_-N loads. The model calibration was conducted manually by adjusting parameter values within an allowable range, following the technical guideline of the SWAT model. Parameter values were selected yielding the best statistical performance measures while satisfying the SWAT performance criteria suggested by Moriasi et al. (2007) [22]. The following statistical performance measures were considered: Nash-Sutcliffe efficiency coefficient (NSE), root mean square error (RMSE), standard deviation ratio (RSR), and percent bias (P-bias), shown as:

$$NSE=1-\left[\frac{\sum_{i=1}^{n}{\left({\text{S}}_{i}\;-{\text{ O}}_{i}\right)}^{2}}{\sum_{i=1}^{n}{\left(\bar{\text{O}}-\;{\text{O}}_{i}\right)}^{2}}\right]$$ (1)

$$RSR=\frac{RMSE}{STDEV_{obs}}=\left[\frac{\sqrt{\sum_{i=1}^{n}{\left({\text{O}}_{i}-{\text{S}}_{i}\right)}^{2}}}{\sqrt{\sum_{i=1}^{n}{\left({\text{O}}_{i}-\bar{\text{O}}\right)}^{2}}}\right]$$ (2)

$$P-bias=\left[\frac{\sum_{i=1}^{n}\left({\text{S}}_{i}-{\text{O}}_{i}\right)\times 100}{\sum_{i=1}^{n}{\text{O}}_{i}}\right]$$ (3)

where O_*i*_ is observed and S_*i*_ simulated data, $\bar{\text{O}}$ is observed mean values, and n equals the number of observations. In addition, the model uncertainty was assessed using the 95 percent prediction uncertainty (95 PPU) range suggested by Singh et al. (2014) [23]. This was computed using all simulation outputs obtained during the manual calibration process. The 95 PPU value was calculated at the 2.5 and 97.5 percentiles of the cumulative distribution of simulation outputs [23].

**Table 4:** The list of calibrated parameters.

Parameter	Description (unit)	Range	Calibrated value	
			TCW	GW
CN2 [24]	Curve number	-50–50%	-30%	0%
ESCO [24]	Soil evaporation compensation factor	0–1	1	0.95*
SURLAG [24]	Surface runoff lag coefficient	0.5–24	0.5	0.5
SOL_AWC [24]	Available water capacity of the soil layer (mm H2O·mm soil^-1^)	-50–50%	- 10%	- 1%
SOL_K [24]	Saturated hydraulic conductivity (mm·hr^-1^)	-50–50%	50%	-50%
SOL_Z [24]	Depth from soil surface to bottom of layer (mm)	-50–50%	-20%	-31%
ALPHA_BF [24]	Base flow recession constant (1·days^-1^)	0–1	0.07	0.051
GW_DELAY [24]	Groundwater delay time (days)	0–500	120	40
GW_REVAP [24]	Groundwater “revap” coefficient	0.02–0.2	0.10	0.02*
RCHRG_DP[24]	Deep aquifer percolation fraction	0–1	0.01	0.01*
GWQMN[24]	Threshold depth of water in the shallow aquifer required for return flow to occur (mm)	0–5000	1.9	1.0
CH_K2 [24]	Effective hydraulic conductivity (mm·hr^-1^)	0–150	0*	25
CH_N2 [24]	Manning coefficient	0.01–0.3	0.29	0.025
NPERCO [9]	Nitrogen percolation coefficient	0.01–1	0.5	0.15
N_UPDIS [9]	Nitrogen uptake distribution parameter	5–50	50	50
ANION_EXCL [9]	Fraction of porosity from which anions are excluded	0.1–0.7	0.59	0.6
ERORGN [9]	Organic N enrichment ratio for loading with sediment	0–5	4.92	4.1
BIOMIX [9]	Biological mixing efficiency	0.01–1	0.01	0.01
SOL_NO3 [25]	Initial NO3 concentration in soil layer (mg N·kg^-1^)	0–100	11.23	0
CDN [17]	Denitrification exponential rate coefficient	0–3.0	0.3	2.9
SDNCO [17]	Denitrification threshold water content	0.1–1.1	1.0	1.0
LAIMX1 [8,9]	Fraction of the maximum leaf area index corresponding to the first point on the leaf area development curve	-	0.01 (Wheat)	
		-	0.02 (Barley)	
		-	0.12 (Rye)	
LAIMX2 [8,9]	Fraction of the maximum leaf area index corresponding to the second point	-	0.14 (Wheat)	
		-	0.31 (Barley)	
		-	0.35 (Rye)	

Note:

*(an asterisk) refers to a default value.

The ranges of parameters were adapted from previous literature (the number within parentheses).

The SWAT parameters affecting summertime crop growth, N fixation, and soil N mineralization were at the default values. However, the parameters related to the growth of WCCs were adjusted to more realistically replicate observed WCC growth in the local region as N uptake by WCC is primarily dependent upon cover crop biomass [8]. We used the method employed by Yeo et al. (2014) [9] to simulate “representative” biomass growth at the field scale per species, considering substantial variation. This method adjusts the plant growth parameters that control the leaf area development curve using the potential heat unit (PHU) theory as implemented in the SWAT to match estimates of biomass (simulated at the HRU scale) with those observed at the field scale as described by Hively et al. (2009) [8]. They reported landscape-level biomass estimates for three commonly used winter cover crop species categorized by various planting dates in the Choptank River region using multi-temporal satellite remote sensing observations and field sampling data (133 sites) over the winter season (Oct.–Mar.) by including relative growth observations for a single winter growth period (2005–2006) [8]. The SWAT cover crop growth was calibrated to produce 7-year average biomass outputs estimated at the HRU scale to be consistent with the 1-year observation. The WCC results, therefore, did not capture any inter-annual variation in biomass and N accumulation resulting from annual climatic conditions. However, this should not considerably affect plant growth simulation even if there is some inter-annual variability in weather conditions during the monitoring period. The plant growth cycle in the SWAT was simulated using the heat unit theory. It predicts plant growth based on the heat unit. Heat units are estimated based on the cumulative daily temperature above the base temperature relative to potential heat units required for the plant maturity. Not only significant changes in cumulative heat units normalized by potential heat units during the monitoring period, but also the small difference in heat units did not produce substantial variation in WCC biomass growth. See Yeo et al. (2014) [9] for further discussion. Note that the simulated WCC biomass outputs were analyzed for 7 years, as there are 7 full winter terms (Oct.–Mar.) over the eight calendar year (Jan.–Dec.) simulation period.

### Statistical Analysis of WCC impacts

Following the SWAT modeling, several statistical analyses were conducted to compare treatment effects (no WCC versus WCCs) on water and NO_3_-N budgets within and between watersheds, using either annually or seasonally (winter season) simulated water and NO_3_-N loads. We first asked if the two watersheds showed significantly different hydrological responses as hypothesized, using a one-way multivariate analysis of variance (MANOVA) and a two-sample t-test. A three way analysis of variance (ANOVA) and the Bonferroni procedure were then used to investigate if the WCC N uptake efficiency varied by site characteristics (i.e., dominant soils in watershed), WCC planting species, WCC planting timing, and their interactions. The effects of cropland soils on NO_3_-N delivery mechanisms were further investigated by performing a one-way ANOVA and the Bonferroni procedure which compared the overall WCC effects on the seasonal NO_3_-N loads against the baseline. Note that the effect of the two crop rotations (i.e., continuous corn and corn-soybean rotation) on WCC performance in NO_3_-N reduction was analyzed only for the early-planted rye (RE) scenario in TCW, which resulted in the greatest reduction in NO_3_-N budgets. A paired sample t-test was used to assess crop rotation effects on NO_3_-N loads as compared to the baseline. As the statistical methods we employed were sensitive to the outliers, we carefully inspected the sample distribution of the hydrological variables and removed outliers prior to statistical testing. An outlier was identified when an observation point fell more than 1.5 times the interquartile range above the 3^rd^ quartile or below the 1^st^ quartile. The annual and seasonal hydrological variables obtained in 2003 were extremely high and thus were determined to be outliers. This was most likely due to the extremely high precipitation that occurred in 2003. The annual and seasonal precipitation in 2003 ranked the highest over the last 30 years.

## Results and Discussion

### SWAT calibration and validation

**Fig 3:**
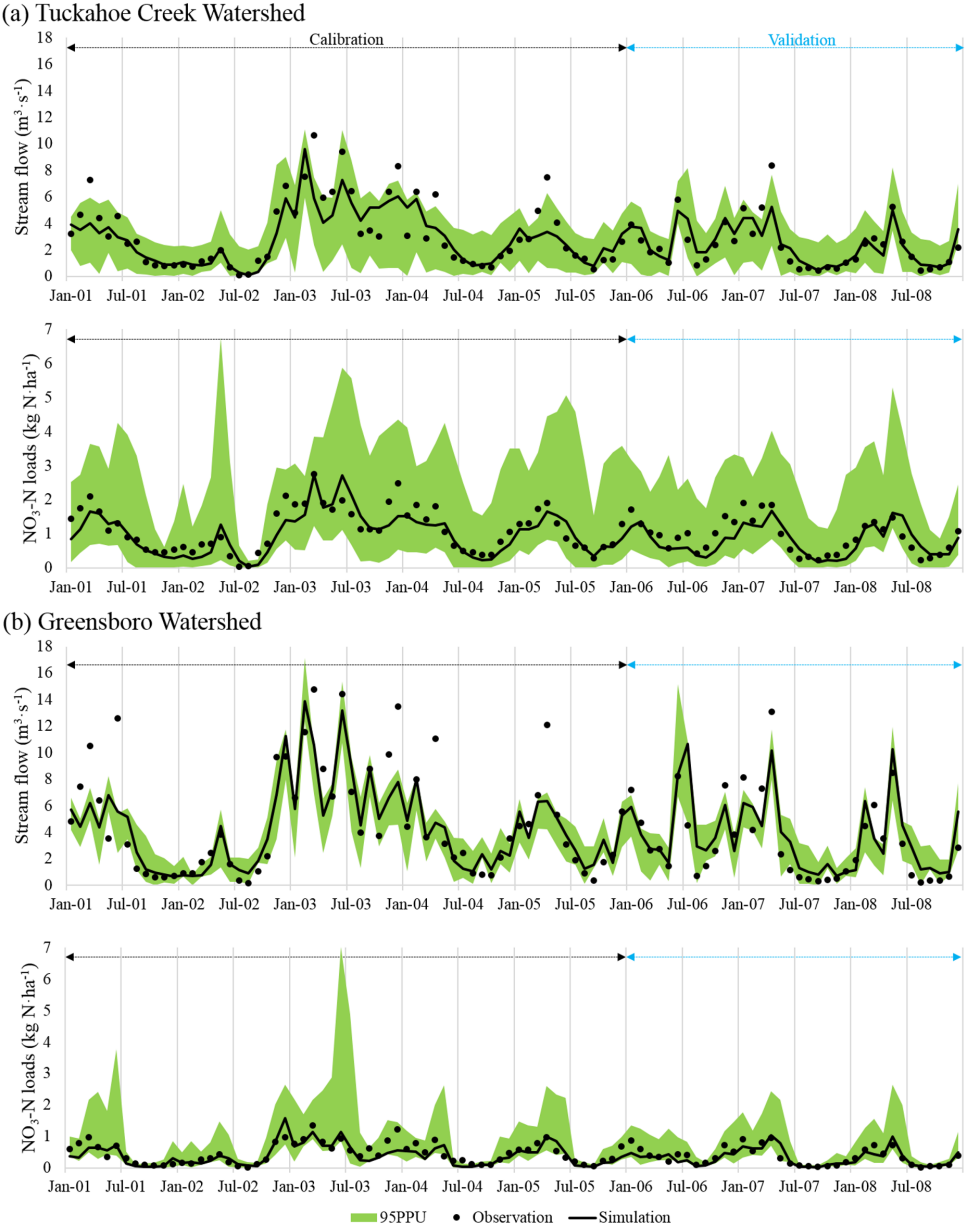
Comparison of observed and simulated monthly stream flow and NO_3_-N loads for the (a) Tuckahoe Creek Watershed and (b) Greensboro Watershed.

Overall, monthly stream flow and NO_3_-N loads simulated from the calibrated model were in good agreement with corresponding observed values (Fig 3). Seasonal variations in both the stream flow and NO_3_-N loads were well depicted by the SWAT simulations. However, the 95 PPU band (shown by interval in Fig 3) did not capture observed peak stream flow for TCW and GW. The CN surface runoff method used in the SWAT limited predicting storm effect, because the duration and intensity of precipitation were not considered in surface runoff calculation. This resulted in underestimation of peak stream flow [26]. Precipitation obtained from remote weather stations (~35 km away from the outlet of the watersheds) may not have provided accurate values for the watershed [27]. Accordingly, localized storm effect might not be reflected in simulations. In addition, poorly simulated ET might increase water loss, resulting in underestimation of stream flow during summer periods [27].

The SWAT underestimated NO_3_-N loads during low-flow seasons, particularly for TCW. Underestimation of NO_3_-N loads during the low-flow seasons in TCW have also been reported by several studies [9, 28, 29]. Previous studies [9, 30] attributed lower estimates of NO_3_-N loads to the underestimation of stream flow, inherent limitation of the SWAT’s capacity to simulate N cycle in lowlands, and inaccurate simulation of summer crop growth relative to N uptake by plants. The residence time of groundwater NO_3_-N in the eastern shore region of the CBW range from a few years to several decades [4]. Fertilizer application rates in the 1970’s to 1990’s were much higher than current rates [15] and 2 years of the model warm-up (1999–2000) might not be sufficient to represent this background NO_3_-N from the past fertilization. Considering long residence time, N fertilizer applied several decades ago could presently be discharged to streams and lead to substantially higher in-stream NO_3_-N concentration, lowering model predictability. Also NO_3_-N loads based on field sampling of stream concentration (i.e., the observed NO_3_-N load) could be inaccurate if field samples were not collected frequently over the long term, leading to the extrapolation of misleading “observed” NO_3_-N loads [9, 31].

Model performance measures and accuracy ratings under the baseline scenario are summarized in Table 5. Accuracy ratings were based on statistical evaluation guidelines from Moriasi et al. (2007) [22]. Overall model calibration and validation results were satisfactory for both watersheds. Simulations for stream flow were better matched with observations than for NO_3_-N loads. Model performances for NO_3_-N loads under the baseline condition were classified as satisfactory, good, and very good (Table 5).

**Table 5:** Model performance measures for stream flow and NO_3_-N loads.

Period	Variable	Stream flow		NO3-N loads	
		TCW	GW	TCW	GW
Calibration	NSE	0.705**	0.703**	0.687**	0.594*
	RSR	0.537**	0.540**	0.554**	0.631*
	P-bias (%)	-9.4***	-9.4***	-10.8***	-13.7***
Validation	NSE	0.759***	0.661**	0.561*	0.631*
	RSR	0.483***	0.573**	0.652*	0.598**
	P-bias (%)	2.7***	12.8***	-12.9***	-9.8***

Note: Model performances were rated based on the criteria of Moriasi et al. (2008) [22];

* Satisfactory,

** Good,

*** Very Good.

**Fig 4:**
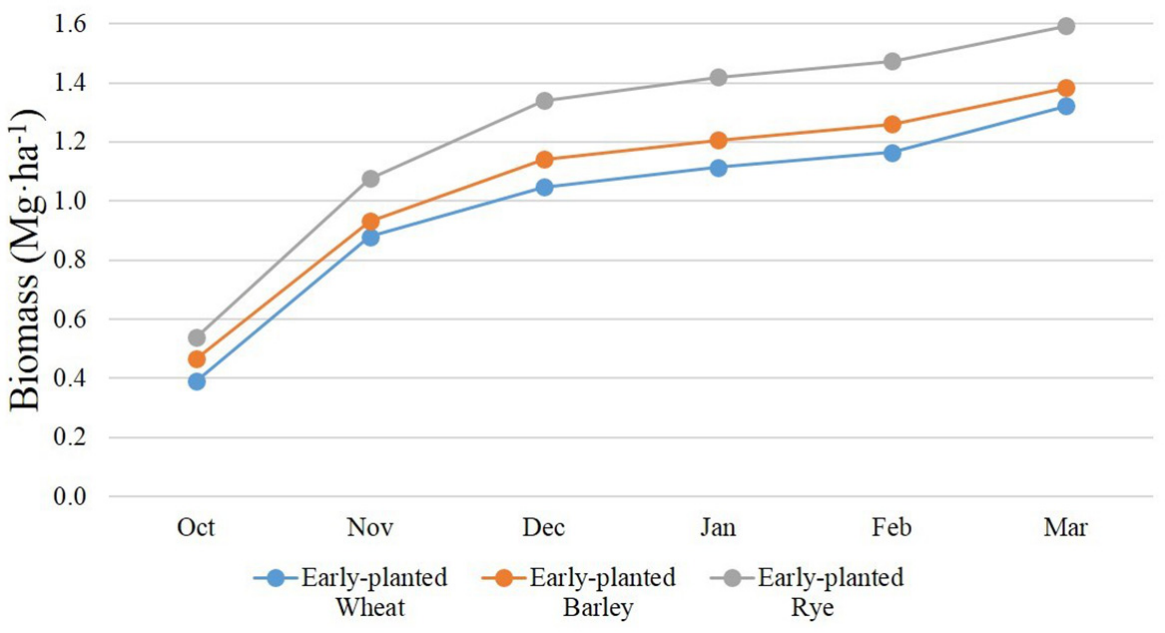
7-year average of early-planted winter cover crop biomass over winter seasons (October–March) calibrated to the field observation collected from 2005–2006.

Nitrate-N reduction by WCCs is achieved by the transformation of soil N into WCC biomass N. Therefore, accurate simulation of WCC biomass accumulation could lead to enhanced prediction of WCC N uptake and its effect on NO_3_-N loads. The WCC growth was calibrated within the SWAT model to match with the actual regional growth pattern in the winter of 2005–2006 following methods used by Hively et al. [8] (Fig 4). Note that in this region rye grows quickly and often has the greater biomass than barley and wheat [8]. In contrast, pre-calibrated simulation outputs (results not shown in this paper) using default SWAT growth parameters would have estimated greater winter wheat and barley biomass than rye biomass because their growth was considered during summer compared to winter growing season. Note that WCC biomass during 2001–2008 was calibrated to the specific remote sensing observations made for 2005–2006 [8]. An inter-annual assessment of WCC biomass could not be undertaken in this study due to data unavailability. However, the method described by Yeo et al. (2014) [9] calibrated data over a limited timeframe (2005–2006) well represented the typical WCC performances in this region. Simulated WCC biomass growth patterns from Yeo et al. (2014) [9] were consistent with field observations made for different years [8, 32, 33]. Furthermore, the estimates of Yeo et al. (2014) [9] were consistent with cover crop N uptake assumed by the Chesapeake Bay Program Watershed Model version 5.3.2 [34]. As the MACS cover crop program collected field WCC biomass data and longer term field observations became available, the WCC biomass calibration can be improved to represent the inter-annual variations in WCC biomass and N uptake under various climate conditions and agricultural practices.

### Watershed-scale assessment of winter cover crop effects on water budget and NO_3_-N loads

**Fig 5:**
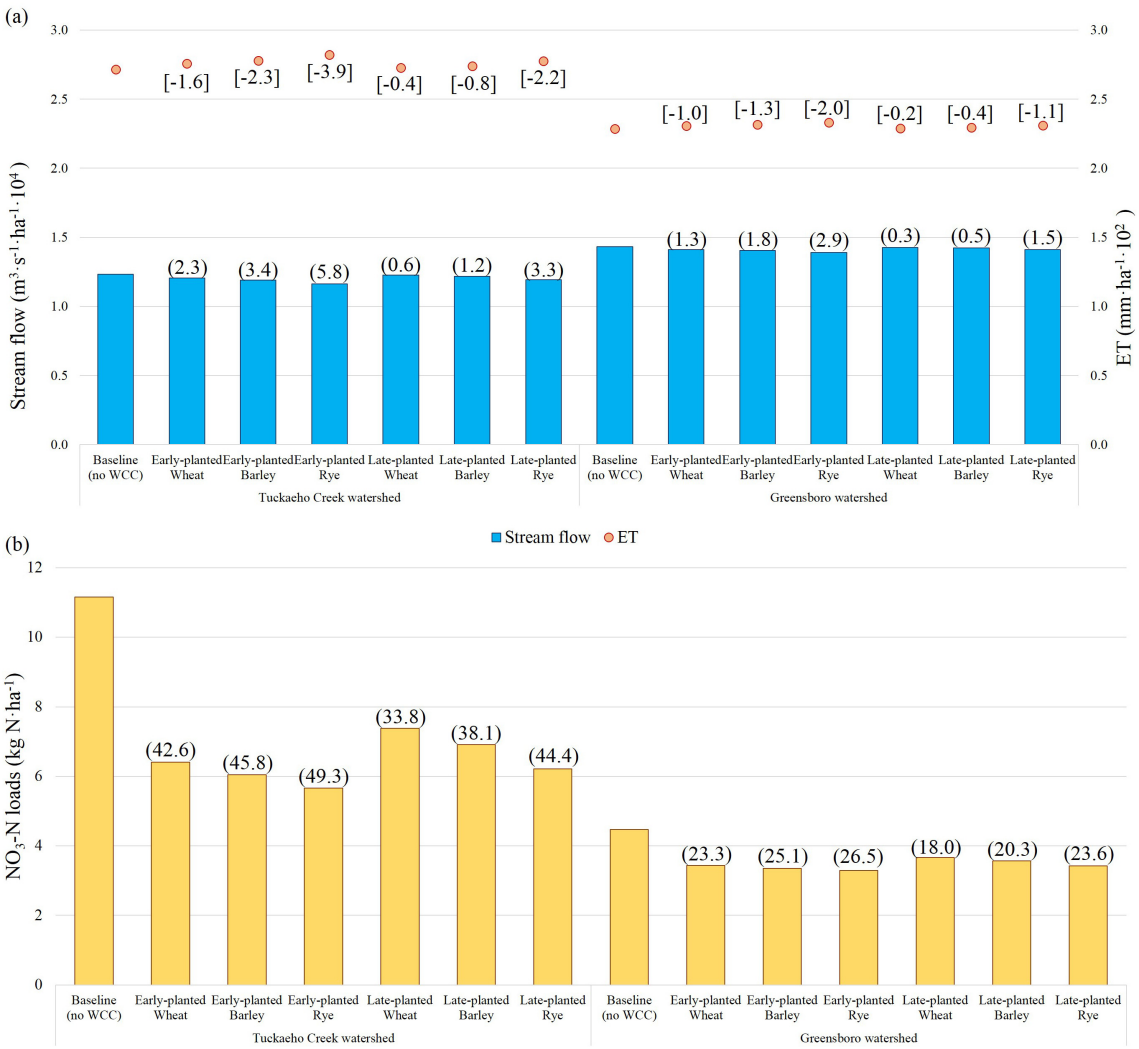
8-year average of annual hydrologic variables under winter cover crop scenarios at the watershed scale: (a) stream flow and evapotranspiration (ET) and (b) NO_3_-N loads normalized by the total watershed area. Note: The numeric values in parentheses ([], (), and ()) indicate reduction rate (RR) of ET, stream flow, and NO_3_-N loads, respectively. RR is calculated by taking the relative difference in simulation outputs from the baseline and WCC scenarios [RR (%) = (Baseline—WCC Scenario) / Baseline × 100]. S3 Table summarizes the finding from the three way ANOVA for the NO_3_-N reduction by the watershed, WCC planting timing, and species.

The 8-year average annual SWAT model outputs were calculated at the watershed scale and summarized by WCC scenarios (Fig 5). Stream flows, ET, and transported NO_3_-N were utilized for the watershed-scale assessment. As hypothesized, the two watersheds showed significantly different hydrological responses (*p-*value < 0.001 from the one-way MANOVA). A previous regional study showed that WCCs had negligible impacts on stream flow and ET, but greatly affected NO_3_-N loads [9]. Similarly, our simulation results also showed small changes in stream flow and ET regardless of WCC implementation for both watersheds. Our findings showed that the slightly greater changes in stream flow and ET were caused by rye compared to barley and wheat due to its relatively higher growth rate and biomass yield. Early planting of cover crops induced a slight increase in ET and decrease in stream flow relative to late planting (Fig 5a).

The impacts of WCCs on NO_3_-N loads were much more noticeable than the effects on stream flow (Fig 5b). Compared to the baseline, annual NO_3_-N loads after WCC treatments were significantly different for the two watersheds (*p-*value < 0.000) by WCC planting timing (*p-*value < 0.013) and species (*p-*value < 0.036) (S3 Table). Annual NO_3_-N loads decreased from 11.2 (Baseline) to 5.7 (RE) kg N·ha^-1^ for TCW and from 4.5 (Baseline) to 3.3 (RE) kg N·ha^-1^ for GW. This is equivalent to NO_3_-N load reductions of 0.9 to 5.8 kg N·ha^-1^ or from 33.8% (WL) to 49.3% (RE) for TCW and from 18.0% (WL) to 26.5% (RE) for GW. Relative to late planting, early-planted WCCs lowered NO_3_-N loads by 0.5 kg N·ha^-1^ on average (95% confidence interval: 0.1 to 0.8 N·ha^-1^). The difference in N uptake by WCC between two planting timings was greater in TCW (8.8%, 0.6–1.0 kg N·ha^-1^), than in GW (5.3%, 0.1–0.2 kg N·ha^-1^). Overall, this occurred most effectively in RE. The longer growing days and warmer conditions for early-planted WCCs promoted growth and biomass and therefore resulted in higher N uptake than late-planted ones [35]. Rye is a hardy species and establishes its root system more rapidly, thereby resulting in greater N uptake than other crops [36]. The TCW showed higher NO_3_-N loads and WCC N uptake compared to GW (95% CI for the differences in NO_3_-N loads between two watersheds: 3.35 to 4.06 kg N·ha^-1^), likely due to larger area of croplands mainly located on well-drained soils.

### Site characteristic impacts on NO_3_-N fate and winter cover crop performance

**Fig 6:**
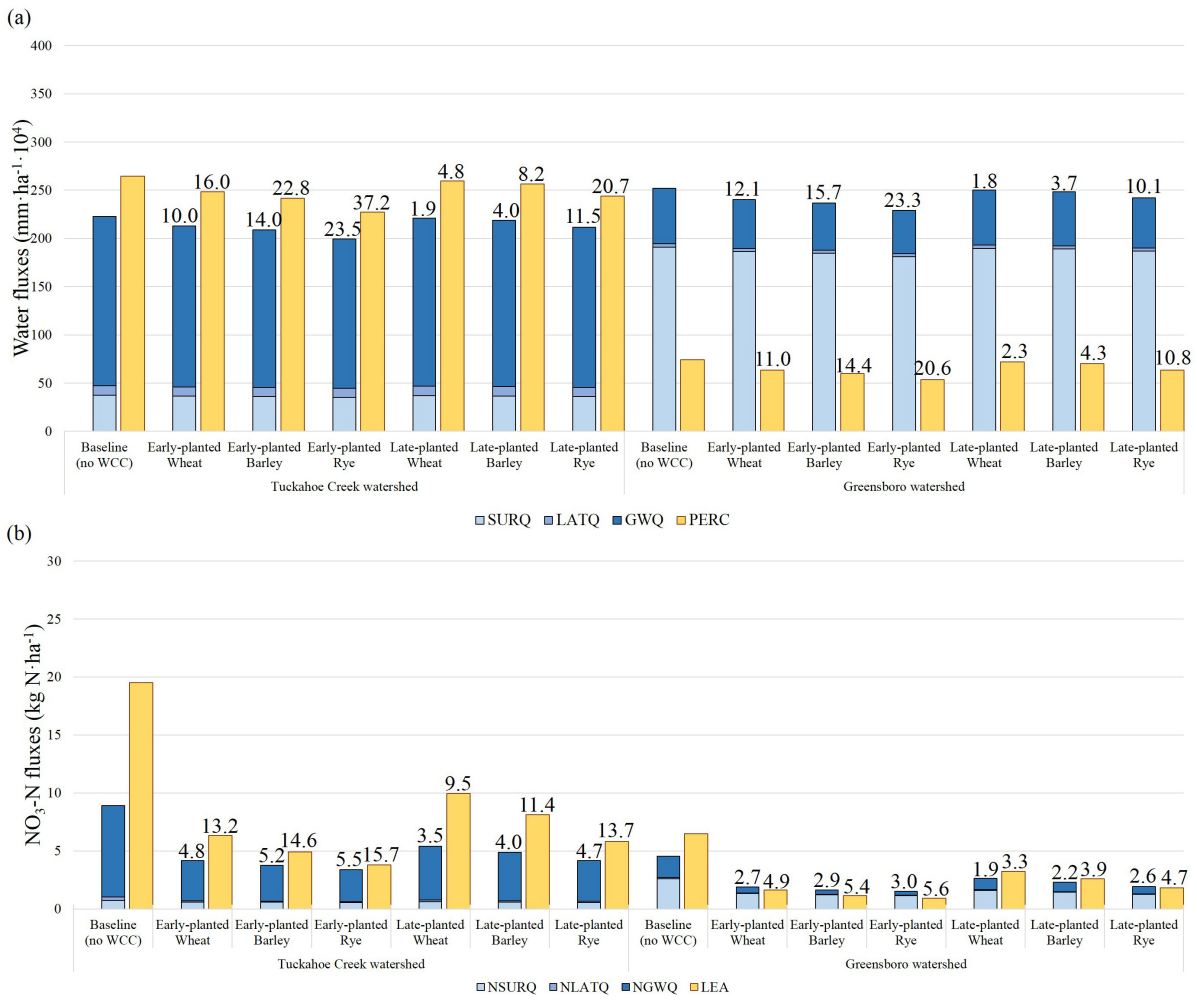
7-year average of annual (a) water and (b) NO_3_-N fluxes at the cropland scale under winter cover crop scenarios over winter seasons (October-March) normalized by the total cropland area. Note: The SURQ, LATQ, and GWQ in (a) refer to water fluxes delivered to streams by surface runoff, lateral flow, and groundwater flow, respectively. The PERC refers to water percolation entering to groundwater from the bottom of the soil profile. The NSURQ, NLATQ, and NGWQ in (b) refer to NO_3_-N fluxes delivered to streams from croplands by surface runoff, lateral flow, and groundwater flow, respectively. The LEA is NO_3_-N leaching to groundwater. The PERC and LEA eventually affect the groundwater contribution to streams and NO_3_-N loadings in the groundwater flow over time, respectively. The numeric values on the top of the bar graph indicate the reduction amount of water and NO_3_-N fluxes under winter cover crop scenarios relative to the baseline scenario. The reduction amount by each pathway is available in S4 Table.

Differences in water and NO_3_-N fluxes and WCC performances between the two watersheds were further explained by the cropland-scale outputs (i.e., cropland HRU), excluding other watershed land use over the fall and winter (Oct. to Mar.) of each year (Fig 6). The outputs described the amount of water and NO_3_-N fluxes 1) delivered to streams by surface runoff and lateral and groundwater flow from croplands and 2) entering the groundwater (e.g., percolation from the bottom of soil profile or NO_3_-N leaching). This partitioning elucidated the overall effects of different soil properties on the fate and transport of NO_3_-N and on WCC performance.

The simulation results illustrated that two watersheds have different water and NO_3_-N delivery mechanisms (*p-*value < 0.000 from the one-way MANOVA). In TCW, croplands are mainly located on well-drained soils with high conductivity (Fig 2 & S1 Table). Under the baseline condition, a large amount of water flux moved into deeper soils, passing the bottom of the soil profile and ~78.8% of water fluxes transported to streams by groundwater flow (Fig 6a). Because NO_3_-N in TCW remains stable in subsurface soils and groundwater under aerobic conditions, high NO_3_-N fluxes entered into groundwater (19.5 kg N·ha^-1^) and delivered to the streams by groundwater flow (8.9 kg N·ha^-1^) (Fig 6b). In contrast, 75.7% of water fluxes delivered to streams from GW croplands was attributed to surface runoff, due at least in part to the abundance of poorly-drained soils and high-density ditch systems, while the subsurface flow contribution to streams (e.g., lateral flow and groundwater flow) was 24.3% (Fig 6a). The anaerobic condition in poorly-drained soils is expected to reduce NO_3_-N export through denitrification. Our simulation results also indicated that the amount of NO_3_-N removed by denitrification was 13.0 kg N·ha^-1^ greater in GW croplands during winter seasons compared to TCW croplands. These hydrologic characteristics in GW croplands might contribute to lowering NO_3_-N leached into groundwater (6.5 kg N·ha^-1^) and NO_3_-N fluxes delivered to streams by groundwater flow (4.5 kg N·ha^-1^) compared to TCW croplands. In both watersheds, NO_3_-N leaching was a dominant transport mechanism. The amount of NO_3_-N leached into groundwater was much higher than the amount delivered to the streams from the croplands under the baseline condition.

Compared to the baseline, WCCs were effective at reducing NO_3_-N fluxes delivered to streams (*p*-value < 0.0001 from the one-way ANOVA) and NO_3_-N fluxes leached to ground water (*p-*value < 0.0000 from the one-way ANOVA) for both watersheds. The WCCs were more effective in TCW croplands than in GW croplands (Fig 6b) (one-sided *p-*value< 0. 0000 from a two-sample t-test). Overall, WCCs were more effective in TCW croplands with increased reduction in NO_3_-N fluxes of ~2.5 kg N·ha^-1^ delivered to streams and ~10.1 kg N·ha^-1^ leached into groundwater compared to GW croplands. Compared to the baseline values, NO_3_-N fluxes delivered to streams were lowered by from 3.5 (WL) to 5.5 (RE) kg N·ha^-1^ in TCW croplands and from 1.9 (WL) to 3.0 (RE) kg N·ha^-1^ in GW croplands. The WCCs reduced water percolation by up to ~37.2 mm·ha^-1^·10^4^ and ~20.6 mm·ha^-1^·10^4^ in TCW and GW croplands, respectively, compared to the baseline scenario (no-WCC). This hydrological effect on percolation, combined with WCC effects on NO_3_-N concentration in soils and groundwater, greatly reduced NO_3_-N leaching for both watersheds, by up to ~15.7 kg N·ha^-1^ and ~5.6 kg N·ha^-1^ for TCW and GW croplands, respectively. Seven-fold greater reduction of NO_3_-N fluxes transported to streams by surface runoff was achieved in GW croplands (~1.4 kg N·ha^-1^) compared to TCW croplands (~0.2 kg N·ha^-1^) (S4 Table). Surface runoff accounted for the majority of water fluxes delivered to streams in GW croplands. Therefore, the WCC impact on reduction of NO_3_-N fluxes delivered to streams by surface runoff was more effective in GW croplands than in TCW croplands. The simulated outputs were in good agreement with field observations [37]. Rye cover crop was shown to reduce NO_3_-N leaching by 70.3–86.1% (S4 Table) and field observations reported N reduction rate by rye cover crop ranging from 60 to 94%. The results indicated that WCCs were more effective in reducing subsurface flow and NO_3_-N leaching than in reducing NO_3_-N losses to surface runoff. They also emphasized the importance of WCC implementation on well-drained agricultural soils, since these soils were shown to have higher NO_3_-N levels and a greater potential for NO_3_-N leaching than poorly-drained agricultural soils.

### Crop rotation impacts on winter cover crop performance over winter seasons

**Fig 7:**
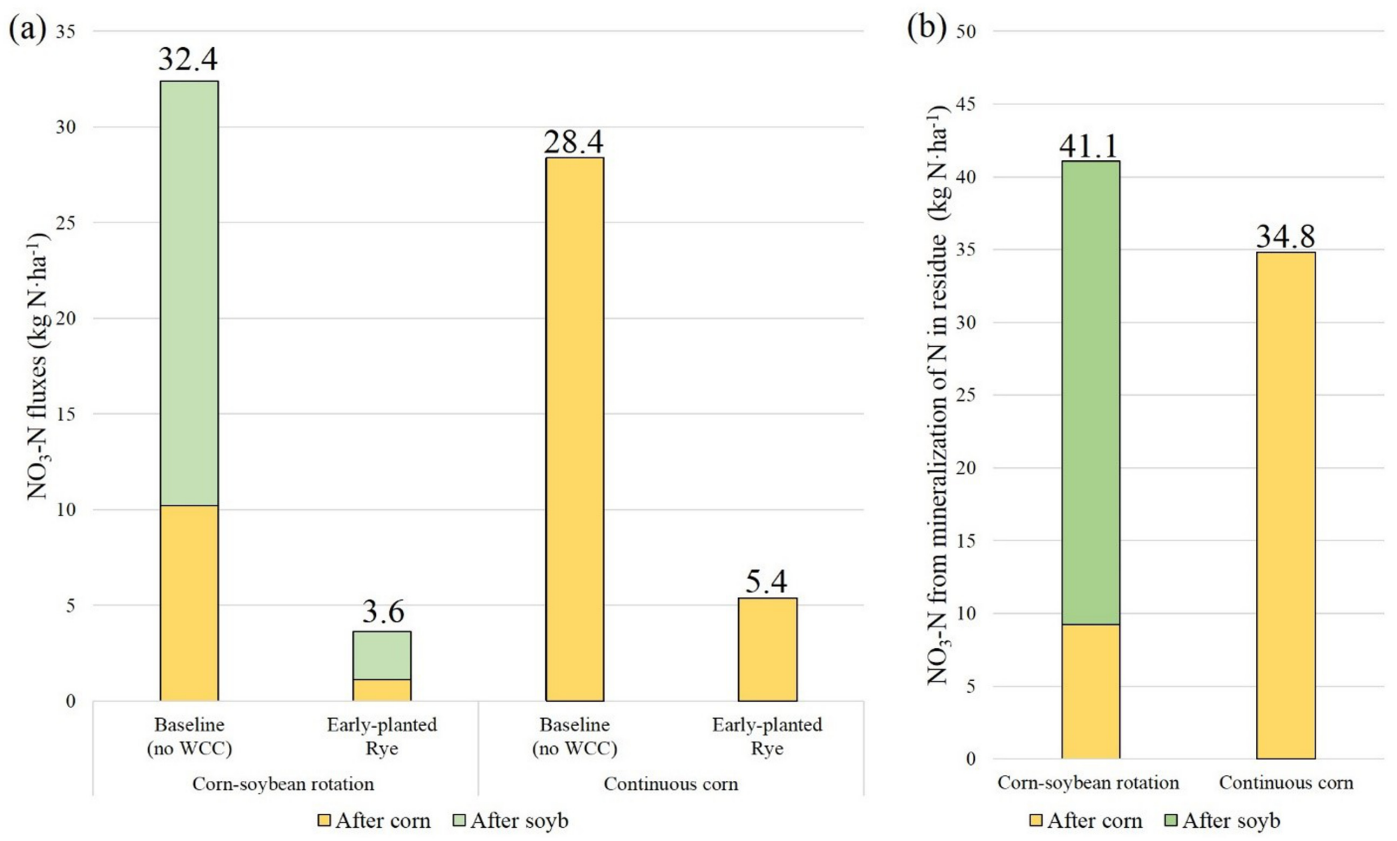
7-year average of annual (a) NO_3_-N fluxes (delivered to streams and leached into groundwater), and (b) NO_3_-N from mineralization of N in residue in two crop rotations at the cropland scale under the baseline and rye early scenarios over winter seasons (October-March). Nitrate-N values are normalized by the total croplands used for these two crop rotations. Monthly values are represented in Fig 8.

**Fig 8:**
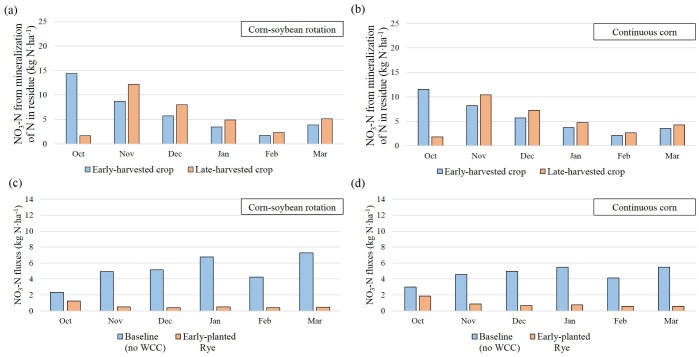
Temporal distribution of 7-year average of (a & b) NO_3_-N from mineralization of N in residue and (c & d) NO_3_-N fluxes (delivered to streams and leached into groundwater) in two crop rotations at the cropland scale under early-planted rye scenario over winter seasons (October-March). Nitrate-N values are normalized by the total croplands used for these two crop rotations. Cumulative values are represented in Fig 7.

Data from the 7-year average annual NO_3_-N fluxes after corn-soybean rotation and continuous corn were compared to evaluate the effects of crop rotations and summer crop species on WCC NO_3_-N uptake. To illustrate differences in the performance of WCC by crop rotations, we used simulation outputs from the RE scenario in TCW, as they had the largest relative reduction rate in comparison to the baseline values. When postharvest NO_3_-N fluxes in two crop rotations were compared under the baseline scenario, corn-soybean rotation exported 4.0 kg N·ha^-1^ more NO_3_-N fluxes than continuous corn (Fig 7a) (one-sided *p*-value = 0.10 from a paired t-test). Nitrate-N fluxes after the harvest of soybean in corn-soybean rotation were higher than after the harvest of corn, accounting for ~68.5% of the total fluxes (Fig 7a). This is likely because soybean often leaves a greater amount of NO_3_-N in the soil due to the rapid rate of residue mineralization [12]. Mineralization is controlled by the C/N ratio of crop residue [38]. As the C/N ratio of residue decreases (i.e., greater N content), the rate of residue mineralization increases [38]. Crops with higher N content (e.g., soybean) typically generate more NO_3_-N than lower N content like corn [11]. Therefore, crop rotations with soybean are expected to have more residual soil N than continuous corn. Our simulation outputs during winter seasons supported these assumptions (Fig 7b). They showed that corn-soybean rotation left 6.3 kg N·ha^-1^ more NO_3_-N from mineralization of N in residue compared to continuous corn. Approximately 77.5% of mineralized N originated from soybean residue in corn-soybean rotation (Fig 7b). The simulation results on postharvest NO_3_-N fluxes after two crop rotations were consistent with the findings from previous observations [39, 40]. Indeed, Fig 8a & 8b shows that mineralized N was greater following earlier harvest than following late harvest when temperatures were relatively high compared to other winter months. This climate condition likely also influenced the amount of mineralized NO_3_-N based on harvesting timing of summer crops (i.e., WCC planting dates). The simulation results showed early-harvested crops (Oct. 1) had substantial N mineralization occurring in October, followed by reduced amounts of mineralization in subsequent months. In contrast, late-harvested crops (Oct. 31) showed highest mineralization in November, with decreased monthly mineralization rates thereafter (Fig 8a & 8b). Overall, the residue from early-harvested crops exhibited approximately 3.7 kg·ha^-1^ more N from mineralization than late-harvested crops over winter seasons.

Because simulated residual mineralized N was substantially greater in soybean than in corn, the efficiency of the RE scenario at reducing NO_3_-N was greater in corn-soybean rotation compared to in continuous corn (one-sided *p-*value = 0.007 from a paired t-test). The RE scenario decreased NO_3_-N fluxes from 32.4 kg N·ha^-1^ to 3.6 kg N·ha^-1^ in corn-soybean rotation and from 28.4 kg N·ha^-1^ to 5.4 kg N·ha^-1^ in continuous corn (Fig 7a). The temporal distribution of NO_3_-N fluxes differed considerably between the baseline and RE scenarios (Fig 8c & 8d). With increasing biomass (Fig 4) and NO_3_-N uptake, a smaller amount of NO_3_-N fluxes was exported to streams and groundwater. This result illustrated the effect of crop rotations on postharvest residual soil NO_3_-N and the WCC NO3-N uptake efficiency. Overall, this result suggested that WCCs may have a greater impact on water quality when planted on fields under crop rotations that produce greater amounts of leachable NO_3_-N.

### Conclusions

A physical model (SWAT) paired with statistical analysis was used to investigate the efficiency of WCCs for reducing NO_3_-N loads under different WCC planting dates, species, soil characteristics, and crop rotations. Overall, the WCCs were more effective when baseline NO_3_-N loads were high due to high leaching potential and/or high availability of residual and mineralized soil NO_3_-N, resulting from soil characteristics and crop rotations. The WCC efficiency varied by planting time and species. Therefore, for the water quality improvements, it is crucial to establish an appropriate WCC treatment depending on local edaphic, hydrologic, and agronomic characteristics. For example, well-drained areas used for frequent cultivation of soybean should adopt a more robust WCC practice (e.g., rye with early planting), while areas with lower infiltration rates, increased denitrification capacity, and lower available soil residual and mineralized N could achieve the same water quality standards with less robust WCC practices (e.g., barley and wheat). The findings of this study can provide key information to aid decision making and to develop effective WCC implementation plans suitable for local characteristics at the watershed scale.

## Supporting Information

S1 Fig The spatial distribution of crop rotations in agricultural fields over the simulation period. Note: Dbl WW/Soyb is described in the caption of Fig 2.
(TIF)

S2 Fig The temporal sequence of crop rotations under the baseline and winter cover crop scenarios. Note: Dbl WW/Soyb is described in the caption of Fig 2.
(TIF)

S1 Table Soil properties and land use distribution of Tuckahoe Creek Watershed (TCW) and Greensboro Watershed (GW). Note: The values in the parenthesis [], denote the proportion of well-drained soils (HSG-A&B) and poorly-drained soils (HSG-C&D) used for agricultural lands, respectively. The explanation on the HSG is available in the caption of Fig 2.
(PDF)

S2 Table Representative crop rotation information and distribution of corn and soybean fields. Note: The last two columns indicate the relative area (%) of each crop rotation applied to croplands in TCW and GW. The bottom four rows indicate the relative area (%) of corn and soybean fields resulted from different rotations applied concurrently in TCW and GW. Dbl WW/Soyb is regarded as soybean fields and described in the caption of Fig 2.
(PDF)

S3 Table Analysis of variance for the reduction of annual NO_3_-N loads by watershed, WCC planting species, and WCC planting timing. (PDF)

S4 Table The reduction amount and rate of water and NO_3_-N fluxes by winter cover crops. Note: The numeric values show the reduction amount and rate (%) of water (mm·ha^-1^) and NO_3_-N fluxes (kg N·ha^-1^), relative to the baseline. The statistical significance for the reduction amount was analyzed with a paired t-test and indicated by following: * *p*-value <0.1; ** <0.05 ***<0.01).
(PDF)

## Abbreviations

WCC: Winter Cover Crop
TCW: Tuckahoe Creek Watershed
GW: Greensboro Watershed
WE: Early-planted Wheat
BE: Early-planted Barley
RE: Early-planted Rye
WL: Late-planted Wheat
BL: Late-planted Barley
RL: Late-planted Rye
Dbl WW/Soyb: Double crops of winter wheat and soybean
SURQ: Water flux delivered to streams by surface runoff
LATQ: Water flux delivered to streams by lateral flow
GWQ: Water flux delivered to streams by groundwater flow
PERC: Water percolation entering to groundwater
NSURQ: NO_3_-N flux delivered to streams by surface runoff
NLATQ: NO_3_-N flux delivered to streams by lateral flow
NGWQ: NO_3_-N flux delivered to streams by groundwater flow
LEA: NO_3_-N leached into groundwater
