# Supplementary figures and images for: Impacts of Watershed Characteristics and Crop Rotations on Winter Cover Crop Nitrate-Nitrogen Uptake Capacity within Agricultural Watersheds in the Chesapeake Bay Region
Source: PLoS One. 2016 Jun 28;11(6):e0157637. doi: 10.1371/journal.pone.0157637 (PMC4924834; doi:10.1371/journal.pone.0157637)

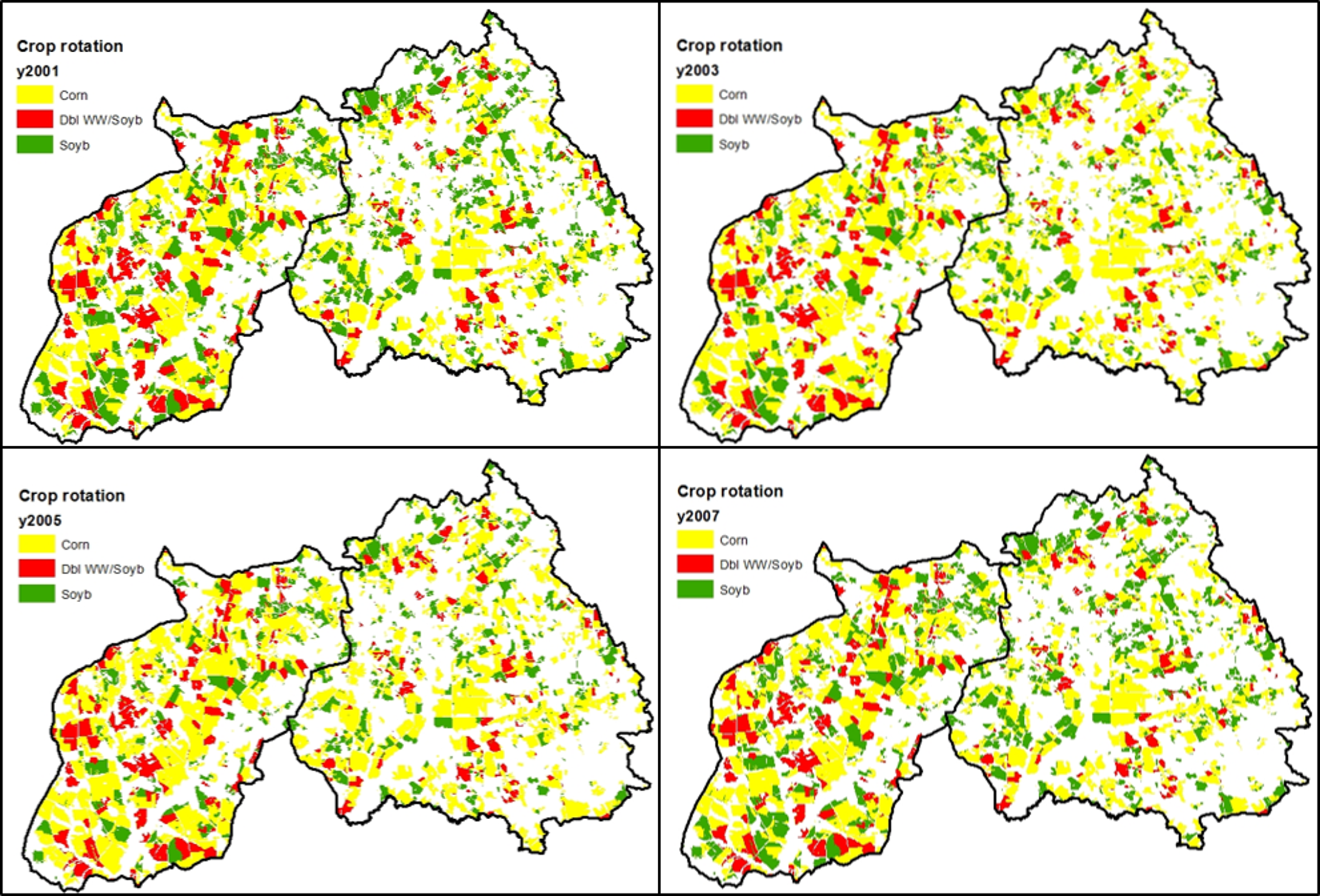

Supplement: S1 Fig — Note: Dbl WW/Soyb is described in the caption of Fig 2. (TIF) [file pone.0157637.s001.tif]
