# Supplementary material for: Impacts of Watershed Characteristics and Crop Rotations on Winter Cover Crop Nitrate-Nitrogen Uptake Capacity within Agricultural Watersheds in the Chesapeake Bay Region
Source: PLoS One. 2016 Jun 28;11(6):e0157637. doi: 10.1371/journal.pone.0157637 (PMC4924834; doi:10.1371/journal.pone.0157637)
Supplement: S1 Table — Note: The values in the parenthesis [], denote the proportion of well-drained soils (HSG-A&B) and poorly-drained soils (HSG-C&D) used for agricultural lands, respectively. The explanation on the HSG is available in the caption of Fig 2. (PDF) [file pone.0157637.s003.pdf]

S1 Table. Soil properties and land use distribution of Tuckahoe Creek Watershed (TCW) and Greensboro Watershed (GW)

Land use	TCW	GW
Agriculture	54.0 % [69.5% / 30.5 %]	36.1 % [32.8% / 67.2 %]
Forest	32.8 %	48.3 %
Pasture	8.4 %	9.3 %
Urban	4.2 %	5.6 %
Water body	0.6 %	0.7 %
**Hydrologic soil groups (HSGs)**	**TCW**	**GW**
A	0.3 %	3.1 %
B	55.8 %	22.4 %
C	2.2 %	4.2 %
D	41.7 %	70.3 %

Note: The values in the parenthesis [], denote the proportion of well-drained soils (HSG-A&B) and poorly-drained soils (HSG-C&D) used for agricultural lands, respectively. The explanation on the HSG is available in the caption of Fig. 2.
