# Supplementary material for: Impacts of Watershed Characteristics and Crop Rotations on Winter Cover Crop Nitrate-Nitrogen Uptake Capacity within Agricultural Watersheds in the Chesapeake Bay Region
Source: PLoS One. 2016 Jun 28;11(6):e0157637. doi: 10.1371/journal.pone.0157637 (PMC4924834; doi:10.1371/journal.pone.0157637)
Supplement: S2 Table — Note: The last two columns indicate the relative area (%) of each crop rotation applied to croplands in TCW and GW. The bottom four rows indicate the relative area (%) of corn and soybean fields resulted from different rotations applied concurrently in TCW and GW. Dbl WW/Soyb is regarded as soybean fields and described in the caption of Fig 2. (PDF) [file pone.0157637.s004.pdf]

S2 Table. Representative crop rotation information and distribution of corn and soybean fields

Type		2001	2002	2003	2004	2005	2006	2007	2008	Proportion (%)	
										TCW	GW
AGA1		Corn	Dbl WW/Soyb	Corn	Dbl WW/Soyb	Corn	Dbl WW/Soyb	Corn	Dbl WW/Soyb	14.5	12.8
AGA2		Dbl WW/Soyb	Corn	Dbl WW/Soyb	Corn	Dbl WW/Soyb	Corn	Dbl WW/Soyb	Corn	21.9	11.1
AGAB		Corn	Soyb	Corn	Dbl WW/Soyb	Corn	Soyb	Corn	Dbl WW/Soyb	7.7	11.6
AGB1		Corn	Soyb	Corn	Soyb	Corn	Soyb	Corn	Soyb	11.3	17.5
AGB2		Soyb	Corn	Soyb	Corn	Soyb	Corn	Soyb	Corn	9.8	10.8
AGC1		Corn	Corn	Corn	Corn	Corn	Corn	Corn	Corn	17.1	10.8
AGD1		Soyb	Corn	Soyb	Soyb	Corn	Soyb	Soyb	Corn	10.2	12.0
AGD2		Soyb	Soyb	Corn	Soyb	Soyb	Corn	Soyb	Soyb	7.5	13.4
TCW	Corn (%)	50.6	59.0	58.1	48.8	60.8	56.3	50.6	59.0	-	-
	Soyb (%)	49.4	41.0	41.9	51.2	39.2	43.7	49.4	41.0	-	-
GW	Corn (%)	52.7	44.7	66.1	32.7	64.7	46.1	52.7	44.7	-	-
	Soyb (%)	47.3	55.3	33.9	67.3	35.3	53.9	47.3	55.3	-	-

Note: The last two columns indicate the relative area (%) of each crop rotation applied to croplands in TCW and GW. The bottom four rows indicates the relative area (%) of corn and soybean fields resulted from different rotations applied concurrently in TCW and GW. Dbl WW/Soyb is regarded as soybean fields and described in the caption of Fig 2.
