# Supplementary material for: Impacts of Watershed Characteristics and Crop Rotations on Winter Cover Crop Nitrate-Nitrogen Uptake Capacity within Agricultural Watersheds in the Chesapeake Bay Region
Source: PLoS One. 2016 Jun 28;11(6):e0157637. doi: 10.1371/journal.pone.0157637 (PMC4924834; doi:10.1371/journal.pone.0157637)
Supplement: S3 Table — (PDF) [file pone.0157637.s005.pdf]

S3 Table. Analysis of variance for the reduction of annual NO_3-N loads by watershed, WCC planting species, and WCC planting timing

Source of Variation	Sum Squares	Degree of freedom	Mean Square	F-Statistics	*p*-Value
**Watershed (Wshd)**	287.939	1	287.939	438.43	**0**
**WCC Planting Species (Species)**	4.576	2	2.288	3.48	**0.036**
**WCC Planting Timing (Timing)**	4.228	1	4.228	6.44	**0.0133**
**Wshd × Species**	2.019	2	1.01	1.54	0.2219
**Wshd × Timing**	1.52	1	1.52	2.31	0.1326
**Species × Timing**	0.212	2	0.106	0.16	0.8513
**Wshd × Species × Timing**	0.082	2	0.041	0.06	0.9397
**Error**	47.286	72	0.657		
**Total**	347.862	83			
