# Supplementary material for: Impacts of Watershed Characteristics and Crop Rotations on Winter Cover Crop Nitrate-Nitrogen Uptake Capacity within Agricultural Watersheds in the Chesapeake Bay Region
Source: PLoS One. 2016 Jun 28;11(6):e0157637. doi: 10.1371/journal.pone.0157637 (PMC4924834; doi:10.1371/journal.pone.0157637)
Supplement: S4 Table — Note: The numeric values show the reduction amount and rate (%) of water (mm·ha-1) and NO3-N fluxes (kg N·ha-1), relative to the baseline. The statistical significance for the reduction amount was analyzed with a paired t-test and indicated by following: * p-value <0.1; ** <0.05 ***<0.01). (PDF) [file pone.0157637.s006.pdf]

S4 Table. The reduction amount and rate of water and NO_3-N fluxes by winter cover crops

Watershed	Scenario	Water fluxes, $mm \cdot ha^{-1} \cdot 10^4$ (%)				NO_3-N fluxes, $kg\ N \cdot ha^{-1}$ (%)			
		SURQ	LATQ	GWQ	PERC	NSURQ	NLATQ	NGWQ	LEA
TCW	Early-planted wheat	0.72 (2.3)	0.37 (4.4)	7.23 (4.9)	13.34 (6)	0.2 (21.7)	0.2 (63.1)	4.4 (56)	13.2 (67.5)
	Early-planted barley	0.98 (3.2)	0.52 (6.2)	10.21 (7)	19.01 (8.6)	0.2 (23.8)	0.2 (70.5)	4.8 (60.6)	14.6 (74.8)
	Early-planted rye	1.56 (5)	0.83 (9.9)	17.22 (11.7)	31.04 (14.1)	0.2 (25.8)	0.2 (73.8)	5.1 (65.1)	15.7 (80.5)
	Late-planted wheat	0.31 (1)	0.11 (1.3)	1.2 (0.8)	4.01 (1.8)	0.1 (16.6)	0.1 (50.3)	3.2 (41)	9.5 (48.9)
	Late-planted barley	0.42 (1.3)	0.19 (2.2)	2.69 (1.8)	6.86 (3.1)	0.1 (19.9)	0.2 (58.7)	3.7 (47.2)	11.4 (58.4)
	Late-planted rye	0.88 (2.8)	0.47 (5.6)	8.23 (5.6)	17.25 (7.8)	0.2 (24.3)	0.2 (67.7)	4.4 (55.5)	13.7 (70.3)
GW	Early-planted wheat	4.4 (2.4)	0.2 (4.5)	7 (12.6)	10.5 (14.7)	1.3 (48.9)	0.1 (64)	1.3 (71.4)	4.9 (74.9)
	Early-planted barley	6 (3.3)	0.2 (6.2)	8.9 (16)	13.9 (19.4)	1.4 (53.5)	0.1 (71.4)	1.4 (78.3)	5.4 (82.4)
	Early-planted rye	9.3 (5.1)	0.3 (8.6)	12.8 (23.1)	19.8 (27.7)	1.4 (55.9)	0.1 (72.6)	1.5 (81.4)	5.6 (86.1)
	Late-planted wheat	1.1 (0.6)	0 (1.2)	0.6 (1.1)	2.2 (3.1)	1 (38.2)	0.1 (53.1)	0.9 (46.9)	3.3 (50.1)
	Late-planted barley	1.8 (1)	0.1 (2.2)	1.7 (3)	4.1 (5.8)	1.1 (44.1)	0.1 (60.5)	1 (55.6)	3.9 (59.9)
	Late-planted rye	4 (2.2)	0.2 (5.5)	5.4 (9.8)	10.4 (14.6)	1.3 (51.6)	0.1 (68.3)	1.2 (66.2)	4.7 (71.8)

Note: The numeric values show the reduction amount and rate (%) of water ($mm \cdot ha^{-1} \cdot 10^4$) and NO_3-N fluxes ($kg\ N \cdot ha^{-1}$), relative to the baseline. Pairwise comparisons (with the Baseline) showed that all reduction amounts were statistically significant (one-sided *p*-value <0.05 from a paired t-test).
